# Embracing uncertainty: medical student perceptions of a pediatric bootcamp developed in response to mandated changes during the pandemic

**DOI:** 10.1186/s12909-022-03471-y

**Published:** 2022-05-21

**Authors:** Brittany Lissinna, Marghalara Rashid, Jessica L. Foulds, Karen L. Forbes

**Affiliations:** Department of Pediatrics, University of Alberta, Edmonton Clinic Health Academy, 11405-87 Avenue, Edmonton, AB T6G 1C9 Canada

**Keywords:** Medical education, Transition, Pediatrics, Bootcamp, COVID-19

## Abstract

**Background:**

The start of the COVID-19 pandemic led to both shortened clinical rotations and consequent loss of embedded formal teaching time. In response to these learning gaps, a novel, virtual pediatric bootcamp was developed to provide a consolidated 3-week learning opportunity for clinical medical students. Pre-clinical students were encouraged but not required to participate, given the suspension of clinical patient experiences for all undergraduate medical learners and the uncertainty of when clinical rotations would resume. This group of students were particularly challenged with adapting their learning in response to the pandemic while also preparing to apply their pre-clinical knowledge to solve clinical problems.

**Methods:**

A qualitative thematic analysis was used for this study. Ten semi-structured phone interviews were conducted with second-year medical students to explore their experiences and perceptions of the pediatric bootcamp. The six phases of thematic analysis proposed by Braun and Clark guided data analysis. To ensure rigour, the three aspects of rigour—credibility, transferability and confirmability were utilized throughout the project.

**Results:**

Qualitative exploration from semi-structured phone interviews of second-year medical students’ perceptions and experiences of this new and unanticipated learning experience revealed four main themes: (a) clinical relevance, describing how students were pushed to think about clinical problems in a new way; (b) timing, which explored conflicts related to competing interests, mental preparedness, and the interval between learning and application; (c) teaching strategies, describing how active learning and interaction were facilitated and challenges that arose; and (d) learning resources, highlighting the curated and accessible resources made available to the students, as well as those resources that learners develop for themselves.

**Conclusions:**

A novel three-week online case-based pediatric bootcamp fostered application of knowledge for clinical reasoning at a time when students were transitioning from preclinical to clinical learning. Students were stretched to balance competing priorities, and the bootcamp curated synchronous and asynchronous learner opportunities while allowing them to reflect on their own learning styles and effective virtual learning strategies. While bootcamps are often used to prepare learners for transitions between clinical stages, our findings suggest the bootcamp format can also facilitate transition from preclinical to clinical roles.

**Supplementary Information:**

The online version contains supplementary material available at 10.1186/s12909-022-03471-y.

## Background

Declaration of the COVID-19 pandemic in early 2020 necessitated sudden changes to undergraduate medical education including the clinical learning environment. Medical students on their clinical rotations across North America were precipitously pulled from these experiences. Undergraduate medical programs were left scrambling to adjust learning for their students and to mitigate students’ inability to learn in clinical settings [1]. The existing literature describing undergraduate medical education curricular changes due to COVID-19 is largely limited to proof of concept reports describing novel interventions for particular areas of medicine and reports of numerical ratings of student satisfaction with these changes [2–6]. Narrative reflections from undergraduate medical learners are lacking, particularly from the cohort of students at the transition point from preclinical to clinical learning.

In response to the pandemic, our students were removed from clinical care for 13 weeks. A limited number of teaching sessions were delivered virtually, and case-based learning resources were made available to students, yet many clerkship students went without structured learning. The lost clinical time necessitated significant changes to clinical rotations. The pediatrics rotation was shortened and dedicated pediatric teaching time was eliminated. In response, a virtual pediatric clerkship bootcamp curriculum was rapidly developed and delivered during the students’ time away from their clinical rotations ahead of their anticipated return.

Preclinical courses were also significantly impacted at the onset of the pandemic, quickly transitioning to online delivery for the remainder of the academic year. The downstream effect of disruptions to clinical rotations would carry forward, impacting this particular cohort of students as they transitioned from preclinical to abbreviated clinical experiences. As such, our pediatric clerkship bootcamp was seen as an opporstunity to concurrently provide these preclinical students with an early introduction to clinical learning.

Our pediatric bootcamp curriculum was an opportunistic learning experience for clinical and preclinical students during a time of uncertainty. We were most interested in the unique experiences of our second-year student cohort, for whom the bootcamp provided an early and unanticipated transition to their pediatric clinical learning. Unlike the clinical students, the preclinical students had no previous clinical exposure prior to participating in the bootcamp. Our study objective was to explore the second-year, preclinical medical students’ perceptions, and experiences of the novel pediatric clerkship bootcamp given their unique stage of training.

## Methods

### Context

Over a three-week period in June 2020, students participated in virtual sessions synchronously or asynchronously at their discretion. Curricular content was organized into daily themes and delivered via three to four hour-long sessions per day. Individual sessions were structured as approaches to clinical presentations in order to set the foundation for how students would approach the evaluation of real pediatric patients (e.g., Approach to Pediatric Seizure, Approach to School Problems, Approach to Lymphadenopathy). While individual lecturers were free to present in their chosen format, emphasis was made to illustrate content using clinical cases. This was done to allow students the opportunity to integrate knowledge from different systems and simulate application to virtual patients. Synchronous participation involved students joining a video call with a live instructor. They had access to the “chat” function on the video conference platform and instructors were encouraged to utilize poll software such as “PollEverywhere” and “Kahoot” to facilitate interaction and dialogue between learners and faculty. Developed resources, included pre-readings and pre-work for application during live sessions in a flipped-classroom format. These resources remained accessible after live sessions providing students with flexibility to meet their personal learning needs through self-directed learning, and for future access including during their pediatrics rotations. Participation in the pediatric bootcamp was not mandatory for second-year students, but all students in the class had access to the materials. Clinical and preclinical students were exposed to the same content with the same preceptors. A formative assessment, completed as a team-based learning session, was conducted midway through the bootcamp to introduce students to clinical decision making (CDM) formatted questions. Although attendance was not mandatory, nor tracked, for the midpoint assessment, using the online assessment system 73 participants of the 169 potential second year students completed the activity synchronously. Overall the preclinical students had lower overall scores. A final, optional, and formative multiple choice and CDM assessment was conducted on the last day of the bootcamp. There were no other formal academic opportunities offered to pre-clinical students at this time and second year students had a 4 to 12 month gap between completion of the bootcamp and starting their pediatrics clinical rotation.

At our institution, the preclinical curriculum is organized based on body systems, with content delivered through lectures as well as small group team-based and case-based learning. Students also participate in a longitudinal program including physical exam, communication, professionalism, and introductory clinical experiences. Pediatrics content is delivered as individual lectures embedded within the larger system blocks. Prior to the COVID-19 pandemic, students had a 3 month break between completion of preclinical coursework and the start of clinical rotations. The administration of this course during the gap in learning opportunities was supported, and advertised, by the local undergraduate medical education leadership.

### Study design

This is a qualitative study investigating second-year students’ experiences of the novel pediatric bootcamp curriculum. Thematic analysis, as proposed by Braun and Clarke [7], was used because it is widely used across a range of epistemologies and research questions [8]. Additionally, it enabled us to gain in-depth knowledge about the experiences of undergraduate medical students during the pediatric bootcamp. The constructivist paradigm underpins our study along with its key concept of truth being dependent on one’s perspective. According to the constructivist paradigm, people construct their knowledge and understanding through experiencing things and reflecting on those experiences [9].

### Participants and recruitment

Ten students out of 169 from the medical class of 2022 at the University of Alberta were recruited through purposeful sampling. Nine of these ten students recruited had participated either synchronously or asynchronously in the bootcamp, while one student who volunteered to participate in the interview did not participate nor had they accessed the materials at the time of the interview. Participant recruitment occurred via email invitation distributed by educational administration to all members of the class of 2022. The email invite contained information about the study and asked students to contact a member of the research team if interested in participating. The first ten students to reach out to the research team were included in the study. All participants provided written consent. The study was approved by the University of Alberta Research Ethics Board.

### Data collection

Data was collected through semi-structured phone interviews to adhere to local public health restrictions. The interviews were interactive in nature and an interview guide was prepared with open-ended questions. Deviations from the guide occurred if the interview was naturally progressing well. All interviews were recorded and conducted by a member of the study team who was not involved in instruction or assessment of the pediatric bootcamp. Phone interviews lasted 20–30 min and were scheduled at the preferred time of each participant. All initial interviews were completed prior to the start of clinical rotations. This timing ensured students were able to recall their recent lived experiences during the bootcamp prior to becoming immersed in new experiences on clinical rotations. Interviews were conducted until sufficiency was reached. This occurred when new data did not produce new insights into the topic being examined. The three follow up interviews were completed after the students had started clinical rotations to further explore some areas and validate data.

### Data analysis

Data collection and analysis was completed through ongoing qualitative design. All interviews were transcribed verbatim and reread/re-checked by two team members (BL and MR) to make sure the content was complete and transcribed accurately. We used six phases of thematic analysis proposed by Braun and Clark [7]: (1) becoming familiar with the data; (2) generating codes; (3) searching for themes; (4) reviewing themes; (5) naming themes; and (6) writing the report. In phase one, all four research team members read and reread the transcripts to immerse themselves with the interviews and build an in-depth understanding of the data. Our research team was composed of a resident, two clinician educators with qualitative research experience and a medical education researcher with qualitative research expertise. In phase two, the four researchers coded transcripts independently. In phase three the codes were re-analyzed to sort the codes into major themes and patterns. In phase four, we reviewed and rigorously evaluated the themes to ensure that the participants’ experiences were clearly represented. This involved conducting a more in-depth analysis of the themes and determining the underlying meanings and assumptions in the data. This process led to questions for which we conducted three follow up interviews to provide clarification. In phase five, we actively named and labeled our themes to ensure they were illustrative in nature and appropriately conveyed participants’ narratives. Phase six involved the completion of this manuscript.

### Trustworthiness

Rigour, also referred to as trustworthiness, is what lends credibility to any research. There are three aspects of rigour—credibility, transferability, and confirmability [10]. In the case of our project, credibility occurred with the accuracy of the data that we are reporting. For this we incorporated follow up interviews to ensure that we were accurately conveying our participants’ voices by asking follow-up questions and clarifications. Confirmability/ transferability is saying the research data does not contain any biases or perspectives that are of the researcher but reflects the data collected. We recognize that our own experience and preconceived notions may affect our interpretation of data. Hence, to maintain confirmability in our study, we exposed our preconceptions during discussions with the team (peer debriefing) and openly acknowledge and reflect on how this might affect our analysis (by practicing reflexivity) [11].

## Results

Four main themes were generated following our analysis: clinical relevance, timing, teaching strategies and learning resources.

### Clinical relevance

Participating medical students, who had finished preclinical work but not yet started clinical rotations, described being pushed to think about problems in a new way. With limited clinical experience, they reported occasional difficulties interpreting what was clinically relevant while identifying that they were being asked to apply the knowledge they had learned as preclinical students to more case-based scenarios. They also acknowledged the bootcamp was an opportunity to consolidate their preclinical learning into more clinical scenarios.

#### Transition to thinking clinically

Participants recognized that the bootcamp required them to think and use their knowledge differently in preparation for transitioning to clinical rotations. Students identified that the bootcamp provided a low-stakes environment where they were able to practice these skills.

*“*I think that is one of the great parts of this bootcamp is they really did put an emphasis on clinical reasoning and learning an approach to common presentations, I guess. Like it was instead of, for example, just giving a lecture on bronchiolitis, it was more. Here’s an approach to a child in respiratory distress or things like that. And they also, they added on this team-based learning, where they allowed us to practice clinical decision making in this environment where it didn’t really matter... it felt new and uncomfortable at first, but I think that it’s going to be beneficial not only for the pediatric clerkship rotation, but all rotations beyond that.” [Interview #8]

“You know, antibiotics always get all mixed up in my head, right. So once you can kind of use them a few times and connect them to different experiences it’s a lot easier. Or even, … knowing which which labs to choose for a given presentation when you’ve never, ever ordered labs was challenging. You know, I did my best and I did well, but I wasn’t confident in that component especially.” [Follow-up Interview #1]

#### Clinical reasoning

The students reported a clear difference in how they were taught in the bootcamp compared with how they had learned during preclinical courses. They acknowledged that clinical reasoning is a difficult skill to develop with limited clinical experience. They also emphasized the importance of learning these skills in a low stakes setting.

“I also really loved the CDM [clinical decision making] questions. I think that’s what they were called. I really liked that they gave us those in again, a very low stakes environment just to see how things are asked and how they’re tested and how you have to think about things. ... I think it just gave good preparation and just like a good idea of like, how to think about questions and how to think about even just medicine in general I guess.” [Interview #1]

“ ...sometimes in preclerkship, we just kind of throw everything at the wall and see what sticks and I think maybe you start to see what’s left on the wall once you actually get on the floor and start doing that. So there were definitely lectures where ... you could see that the active clerks were more engaged in the chat and at the same time, you’re hoping that they’re actively involved because they’ve had more exposure. And it’s not just you deficient in that knowledge area. But I expect that that explains part of it, it’s just kind of our maybe lack of complete exposure to all of this.” [Interview #5]

#### Right time for consolidation

Completing bootcamp between preclinical and clinical rotations allowed participants to consolidate their knowledge before the start of their clinical rotation. Participants found this timing helpful as it facilitated a better understanding of the pediatric content they had covered in preclinical courses.

“But just a lot of the material we actually did cover in our first two years. But again, it was kind of in those one or two peds lectures per block kind of scattered throughout. So it was nice having a kind of consolidated review of everything at this bootcamp, while also teaching us a few new things as well as some kind of clinical pearls here and there as well.” [Interview #8]

“I think it gives us kind of a stepping stone to better understanding clinical concepts or content, I think definitely the most learning is still going to be when we’re doing our clerkship rotation. But this was kind of a good bridge between pre clerkship and clerkship if that makes sense.” [Interview #9]

### Timing

Participants described various perspectives related to the timing of the bootcamp. Given our students had just completed their second year of study and would not start their core clinical rotations until four months later, there were key issues related to the unanticipated addition of the bootcamp at the completion of their second year.

#### Competing interests

Some students described having had other planned commitments during the period of time that limited their ability to participate fully in the scheduled learning sessions, or, contrarily, that were compromised because they chose to participate in learning.

“...not convenient for doing other things and I ended up basically those three weeks, I wasn’t able to do my research and wasn’t able to do other things.” [Interview #2]

“I think a big part of it, too, was just that we hadn’t really known what the bootcamp would entail before. So like, we had other things going on in the summer, too. So just me personally, I was moving in the midst of it, too. Which if I had known, just kind of how structured the schedule was, and how much there was, then I probably wouldn’t have moved mid bootcamp.” [Follow-up Interview #3]

Furthermore, some students were frustrated by the impact on their planned summer break, while others appreciated having the learning opportunity to fill the time in advance of their rotation.

“You know, we didn’t get a very long summer. So I was kind of bummed. And that’s part of why it got pushed off was because I’m like, I don’t want to give up three weeks of my two months of summer to work on this.” [Interview #7]

“I think that, especially given that there were not really other learning opportunities at the time. That like, both the, like, being able to accomplish something, and knowing that accomplishing that task would lighten the load later on, I think, was the main reasons.” [Follow-up Interview #2]

“...a lot of the lectures were focused, like in a section of the day it kind of let us Yeah, kind of have a little bit of a summer or enjoy some of the nicer weather as it approached, while still kind of accomplishing some learning.” [Interview #5]

#### Feeling not mentally prepared

Many students describe struggling with an unanticipated change in schedule, causing them to feel less motivated to participate and learn. The timing of the bootcamp resulted in extending their learning into what would have otherwise been a break from studies. Students also describe the mental stress of balancing the pandemic with their studies and other commitments.

“I feel like in my situation, like I was interested in it, because I had that pediatrics elective canceled and this was coming up. But also I felt sort of done for the school year. So I felt I wasn’t giving my full hundred percent effort for it because it was sort of something that we just tacked on to the school year.” [Interview #2]

“It was one week after we finished our competency exam in the middle of a global pandemic. And it was just way too much to handle to also do pediatrics at the same time. ...I had a summer research position lined up that’s a full time position. So I really had no time to do it. No headspace to do it.” [Interview #6]

#### Interval between learning and application

Students identified the interval of time between the bootcamp and their future pediatric clinical rotation as a potential challenge. Students would experience an interval of 4 to 12 months between the end of the bootcamp and their scheduled pediatric clinical rotation; for some they felt the ability to apply their learning to real patients would be difficult and that they would have to relearn the material once they were in the clinical environment. Others appreciated consolidating pediatric content after their first two years.

“Well, I think pediatrics is so different from adult medicine as they always say just because, you know, even vital signs for different age groups are completely different. So I think, you know, when you’re seeing, when I’m seeing kids one day for my pediatric rotation, it’s unfortunately not till next summer, so I’m gonna have to study all of this again.” [Interview #1]

“...it’s a while before my pediatrics block, it’s not till next year sometime. So, I don’t know, things that make information stick for me are like, seeing cases for sure in real life…” [Interview #2]

### Teaching strategies

Participants identified numerous efforts to foster interaction in the virtual bootcamp format. Students recognized successes and challenges associated with these strategies and the variety of platforms and techniques.

#### Facilitating interaction

Students noticed the teaching staff trying their best to create an interactive environment, with the use of audience response systems like PollEverywhere, online game-based learning platforms, like Kahoot, polling functions within the video conferencing design or the chat box functions. Students appreciated different methods to reply, notably that the polling options/audience response systems allowed for anonymity as their username would be attached to the comments whereas comments in the chatbot recorded their username and remained in the chat history. Having an additional facilitator monitoring the chat box allowed medical students to have their questions answered promptly and engage with the ongoing content in real time.

“I think [multiple platforms] facilitated more discussion, especially for many of us who were using Zoom for lecture style things for the first time. It was always like the same handful of people kind of speaking up and stuff like that. But when they introduced PollEverywhere, and Kahoot I think it helps people participate more without like seeing their name displayed on the on the Zoom chat or whatever it might be.” [Interview #4]

#### Challenges with interaction

Sharing perspectives or answers to the questions with the chat function required the students to have confidence in their answers. This was, at times, a barrier to their participation as there were multilevel learners with varied experiences with pediatric medicine, and they were hesitant to be wrong.

“Yeah, the chat box only if I was like really confident in my answer would I say something. Just because it’s not anonymous, so if I answer wrong, like everyone can see that I answered wrong. Which I know like most people don’t care, but it’s just like, you know, you don’t want to be wrong.” [Interview #1]

Students also noted some norms from in-person lectures. Typically, there would not be a place for 100 people to answer a question, so if one person responded with their planned answer in the chat box, they were more reluctant to add their response. Yet, they would answer on the audience response/polling systems. There were also times where the use of additional technology/modalities to participate was distracting or prohibitive to learning.

“The polls required opening the app too and by the time I’d put in the kind of code for it, and then input an answer, I found that I was missing some of the other things that were being said, whereas with the chat box, it didn’t take away from learning what was still going on as well.” [Interview #9]

“...but when they had too many platforms going all at once. It felt like the lecture wasn’t really happening. It was just like us trying to manage the things that were happening on all the different platforms.” [Interview #4]

Active learning strategies in large group sessions were commented on, but the students did miss in-person interactions and the frequent, in-person, small group, team-based or problem-based sessions they were accustomed to from their first two years of undergraduate medical education:

“I think the biggest barrier just has to be that it was over zoom. And I know there were some positives like I’d mentioned earlier about the chat function, etc. But, again, it is a little harder to be totally focused all the time. If you’ve got four hours straight of zoom lectures, and I know I can just stand up and go grab a coffee or whatever and it’s just it that is a little bit harder not having that face to face discussion and interaction with everyone.” [Interview #8]

### Learning resources

Another major theme that emerged from our data was related to learning resources. Our participants reported that the bootcamp created virtual learning resources all in one place for students who could use them at their convenience.

#### Curated and accessible resources

This rotation not only allowed students to have exposure to resources that they could access at any time when needed but also provided core knowledge for pediatrics.

“I guess just having those three weeks dedicated to pediatrics made me feel or it makes me feel more comfortable going into my peds rotation this year because I have those notes I can refer to and they gave us a lot of great resources that I know I can turn to as well.” [Interview #8]

“it gives a lot of kind of, I would say foundational knowledge for pediatrics. Which, if we didn’t have that, I assume we’d be learning a lot of this stuff on our own. I’m not really sure how like the pediatrics rotation worked before COVID. Like it was nice just to have this as a resource as to common problems in pediatrics.” [Interview #9]

Additionally, students also reported that the content of bootcamp were not only accessible but also curated and easy to navigate.

“...And actually I just reminded myself of the, there was a few resources that I found a little more maybe user friendly or or I don’t know, bright is the right word to use, but like the PedsCases podcasts, I think you call it were, yeah, I guess, maybe a little bit more concise and easy to explore.” [Interview #5]

#### What learners developed for themselves

Further, the virtual nature of the pediatric bootcamp created an opportunity for learners to develop their own resources*.* Study participants reported that the bootcamp enabled them to create personalized resources that they could use and refer to when needed.

“I think it’s great because now I have all that information. You know, on my computer, I’ve made the notes I’ve gone through, so it’ll come back quicker.” [Interview #7]

## Discussion

Our study revealed numerous key findings related to second year medical students’ experiences with a virtual pediatric bootcamp taught during the pandemic. Through thematic analysis, four main themes emerged relating to clinical relevance, participants’ perspective on the timing of the boot camp, teaching strategies for fostering interactions while teaching virtually, and development of learning resources. This study adds a unique perspective as we more deeply explored the experiences of those students faced with the transition from preclinical to clinical learning in a time where education delivery was rapidly changing. Our virtual pediatric bootcamp created opportunities for students to begin their transition to clinical thinking, skills transferable beyond just pediatrics, prior to starting clerkship.

The onset of the pandemic and shifts to medical education occurred at the transition between preclinical and clinical stages for second year medical students at our institution. This transition presents medical students with many challenges, including adjusting to learning in clinical settings, applying knowledge to clinical situations and developing their identity in the health care team [12–14]. Transition courses or “bootcamps” have been developed to help prepare students for this important step. Our bootcamp was developed to both supplement lost learning time and provide transitional knowledge and training to second year medical students. A recent scoping review of transition courses designed to promote independent intern practice highlighted the strong evidence for experiential learning and the value of repeated experiences to build on understanding principles [15]. Loss of clinical experience due to the pandemic emphasized the importance of creating a bootcamp that focused on clinical relevance and allowed students to apply preclinical knowledge to patients. The restrictions that were put in place fortuitously allowed us to deliver our bootcamp at a time, perceived by students to be the right time, to consolidate their preclinical teaching.

Our participants provided more insight into their experiences than previous reports of adaptations have offered, including previously reported pediatric bootcamps pulled together with minimal notice. Although reported studies have the same level of the Kirkpatrick [16] model of learner evaluation reaction, our study offers depth of description, explored with interviews and follow-up interviews. While other publications have identified the administrative difficulties scrambling to shift online [3, 17], we were able to show that, although appreciated by the medical students, the impromptu timing was also a challenge for them. Sudden adjustment to learning in the pandemic required students to balance previously planned commitments with virtual learning and navigate the added stress and uncertainty brought on by the pandemic.

Two groups have previously reported that synchronous and asynchronous remote bootcamps or transition courses are feasible for supporting the transition between medical school and residency/internship [18, 19]. Our study adds to the literature in that our bootcamp focused on the transitional stage from preclinical to clinical in a low-stakes environment. Synchronous delivery with an asynchronous learning platform also provided opportunities for students to curate resources they will be able to access later in their training. These findings are in line with current literature which revealed that students reported great satisfaction with the availability of electronic resources offered by virtual learning platforms [20].

Numerous studies in the existing literature related to virtual teaching suggest that one of the main disadvantages of teaching virtually is a tremendous lack of interaction [20, 21]. In our study, participants reported that their preceptors made a huge effort to create an interactive environment while teaching the bootcamp. Virtual synchronous learning was a novel delivery method for undergraduate medical education at our institution and our participants identified several benefits of the new format. The chat function allowed for student participation, educators to audit the chat and answer questions or pose questions to the presenter, and as Garg and colleagues [22] also noted, allowed students to answer their peer’s questions or share key references, elevating the discussion. Other audience response systems like PollEverywhere enhanced engagement with learning material while providing anonymity for the respondents. The virtual platform also created flexibility for participating students and led to the creation of a more diverse set of learning resources [23]. The importance of a psychologically safe learning environment that allows for and welcomes uncertainty and ambiguity are essential to learner wellbeing [24]. Our participants noted that polling the audience allowed for anonymity and safety from others knowing if they were wrong, and this fear of being wrong persisted in a virtual learning environment.

Learning to apply preclinical knowledge to clinical situations and building a scaffold for clinical reasoning skills is a difficult process that requires students to make changes in their learning styles while also navigating the healthcare team and developing new clinical skills [13]. The timing of our bootcamp created an opportunity for students to consolidate their preclinical knowledge through clinical cases and begin shifting their thinking to a more clinical approach in a low-stakes environment before addressing the additional stressors of clinical work. We anticipated students would report feeling overwhelmed with the adjustment to clinical reasoning, but this did not come through, even after follow up interviews where we explicitly asked about perceptions of the workload. This also raises the possibility of more broadly delivering the same curricular content to different levels of learners, and the use of asynchronous or synchronous methods depending on the clinical experiences of the respective learners. Beyond this bootcamp, it could be valuable to explore the opportunities afforded by dual delivery to different stages of learners.

As emphasized by Atherley et al [25], simply focusing on student “preparedness” for clinical work is unrealistic, as we cannot expect students to learn all of the knowledge and skills required to function clinically before their rotations. Rather, transitions should be viewed as a dynamic process with students growing over time. Our participants noted this as well, finding that the clinical reasoning skills they were developing while consolidating pediatric content would be transferable to other clinical rotations and as they gained more experience, they would be able to better apply these skills.

## Strengths and limitations

These study findings are novel and valuable evidence regarding undergraduate medical students’ experiences with pediatric bootcamp at the transitional stage between preclinical and clinical experiences. Our thematic analysis was rigorous, and within our data strong patterns emerged as apparent from the numerous rich students’ quotations. Despite these strengths, our study has several limitations. This study is limited in that we interviewed medical students from a single center who participated in their local pediatric bootcamp and represented only their opinions and perspectives. It is unclear how the perspectives of students would change were they not faced with the uncertainty and novelty of a pandemic while participating in the bootcamp.

## Conclusion

With limited time to prepare, a novel three-week, online, case-based pediatric course fostered application of knowledge for clinical reasoning, stretched learners to balance competing priorities, curated synchronous and asynchronous learner opportunities while allowing them to reflect on their own learning styles and effective virtual learning strategies. Condensed formats during orientation to clinical rotations could provide opportunities for this curriculum to be used moving forward.

## Supplementary Information

**Additional file 1.** 

## Data Availability

The datasets used and/or analysed during the current study are available from the corresponding author on reasonable request.

## Abbreviation

CDM: Clinical decision making
