# Supplementary material for: Embracing uncertainty: medical student perceptions of a pediatric bootcamp developed in response to mandated changes during the pandemic
Source: BMC Med Educ. 2022 May 21;22:390. doi: 10.1186/s12909-022-03471-y (PMC9123158; doi:10.1186/s12909-022-03471-y)
Supplement: Supplementary file 1 — Additional file 1. [file 12909_2022_3471_MOESM1_ESM.docx]

**Appendix:**

**Interview Guide:**

1. Tell me a bit about your experiences in pediatric bootcamp.
2. What did you like about the bootcamp? And why? Please give examples
3. What did you not like? And why? Give examples
4. Did you attend the class regularly?
   1. If so why?
   2. If not, why not?
5. Can you tell me about the class content and material that was taught? Was it helpful? If so, give examples.
   1. If not, what can be done to make it better?
   2. Was the class content overwhelming? If so, why did you feel like that?
6. What did you find to be the biggest barrier with this class?
7. If you attended the sessions, did you participate in the chat box or polls? Why or why not?
   1. Were there any barriers to doing so?
8. Outside of the bootcamp did you review the materials/vodcasts on your own? Why/why not? What are the differences between reviewing on your own versus live?
9. Based on your bootcamp experiences, do you have any insights into your own learning?
10. How was the way material presented during the bootcamp similar or different to your preclinical classes? How did differences impact your learning?
11. Did the way things were presented in the bootcamp impact or change the way you think about how to approach patients or clinical problems? If so, in what way(s)?
12. At the time of the bootcamp you had not begun your clerkship experiences. What impact do you think this (not having had clerkship experiences) had on your ability to learn and understand the clinical content?
13. Any thoughts on how bootcamp could have been improved?
14. Is there anything we have not talked about in our conversation that you would like to talk about?

**Follow Up Interview Guide:**

1. Why was participating in pediatric bootcamp a priority for you?
2. At the time, going through pediatric bootcamp, did you feel it was information overload? Why or why not?

**Pediatric Bootcamp: Session Schedule**

| **Day** | **Theme** | **Session** |
| --- | --- | --- |
| 1 | The Basics | Orientation to Bootcamp and Welcome  Evidence Based Medicine Orientation  Failure to Thrive  Fluids and Hydration  Pain Management in Children |
| 2 | Babies | Newborn Physical Exam  Neonatal Sepsis  Approach to the Preterm Baby  Neonatal Jaundice |
| 3 | Respiratory | Neonatal Respiratory Distress  Approach to Respiratory Distress |
| 4 | Cardiology | Approach to Murmurs  Approach to the Blue Baby (Cyanotic CHD)  Acyantotic CHD and Acquired Heart Conditions |
| 5 | MSK/Dermatology | Common Skin Conditions in the Newborn & Child  Approach to the Limping Child |
| 6 | Neurology | Approach to Pediatric Seizures  Approach to Pediatric Headache  Approach to Paroxysmal Events |
| 7 | Acutely Ill Child | Fever/Meningitis  Emergency - Pediatric Trauma  Emergency – The Unstable Child |
| 8 | Midpoint Review | Journal Club #1 (Therapy)  Radiology Review  Clinical Decision Making Team Based Learning |
| 9 | GI and Nutrition | Nutrition and Healthy Active Living  Approach to Vomiting  Approach to Diarrhea |
| 10 | Preventative Health/Health Promotion | Child Abuse  Safety and Injury Prevention  Introduction to Adolescent Medicine |
| 11 | Developmental Pediatrics | Child Development  Approach to School Problems  Child with Disabilities |
| 12 | Hematology/Oncology | Approach to Bleeding and Bruising  Approach to Anemia  Approach to Lymphadenopathy |
| 13 | ENT and Kidneys | Approach to Pediatric ENT Infections  Approach to Edema  Approach to Hematuria |
| 14 | Review | Journal Club #2 (Meta-analysis)  Growth and Puberty Review  Infectious Disease Review/Consolidation |
| 15 | MCQ | Optional/Formative |
